# A rare case of highly differentiated follicular carcinoma in ovary with FGFR4 Gly388Arg polymorphism: a case report and literature review

**DOI:** 10.1186/s13048-022-01007-y

**Published:** 2022-06-14

**Authors:** Yi-Ting Bao, Chao Wang, Wu Huang, Liang-Qing Yao, Lei Yuan

**Affiliations:** 1 Department of Gynecologic Oncology, Obstetrics and Gynecology Hospital of Fudan University, Shanghai, China; 2 Department of Pathology, Obstetrics and Gynecology Hospital of Fudan University, Shanghai, China

**Keywords:** Highly differentiated follicular carcinoma (HDFCO), Malignant struma ovarii, FGFR4 Gly388Arg, Mutation

## Abstract

**Background:**

Highly differentiated follicular carcinoma (HDFCO) is a rare form of struma-derived thyroid-type carcinoma in ovary, defined as ovarian struma spreading beyond ovary but consisting of benign thyroid tissues. No more than 30 cases of HDFCO have been reported since it was first recognized in 2008. The clinicopathologic and molecular features of HDFCO remain unclear up till now.

**Case presentation:**

A 38-year-old, para 1 gravida 5 woman has a long history of recurrent right ovarian cysts. Histological evaluation showed the tumor progressed from ovarian mature cystic teratoma (OMCT) to highly differentiated follicular carcinoma (HDFCO) during three relapses. Whole-exome sequencing revealed the germline FGFR4 Gly388Arg polymorphism. Repeated operations were performed to remove lesions for the first two relapses. On the third recurrence, the patient received radical surgery with subsequent thyroidectomy and radioactive iodine ablation. No evidence of disease was observed by February 2022 (8 months).

**Conclusions:**

The germline FGFR4 Gly388Arg polymorphism may accelerate the malignant transformation of HDFCO, probably by working as a second hit in the developing spectrum.

**Supplementary Information:**

The online version contains supplementary material available at 10.1186/s13048-022-01007-y.

## Background

Highly differentiated follicular carcinoma (HDFCO) of ovarian origin is a form of struma-derived thyroid-type carcinoma, defined as ovarian struma spreading beyond ovary but consisting of benign thyroid tissues [1]. No more than 30 cases of HDFCO have been reported since it was first recognized in 2008. The clinicopathologic and molecular features of HDFCO have been gradually studied this decade but remain unclear up till now. It usually arises from a struma ovarii (SO), less frequently associated with ovarian mature cystic teratomas (OMCT) [2]. It is biologically malignant but well differentiated with a good prognosis, which makes the treatment controversial. No standard treatment exists at present, including local resection of lesions with or without subsequent thyroidectomy and radioactive iodine ablation [1]. HDFCO shares little similarity with typical cervical thyroid cancer and struma-derived carcinoma in gene aberrations. The mechanism of extraovarian spread and malignant conversion is still under exploration.

Herein, we presented a much more aggressive case than reported, which harbored FGFR4 mutation and showed the consecutive transformation from OMCT to HDFCO. By histological evaluation and molecular analysis, we drew a developing spectrum of HDFCO and explored potential functions of FGFR4 Gly388Arg alteration in the tumor development.

### Patient with HDFCO

**Fig. 1:**
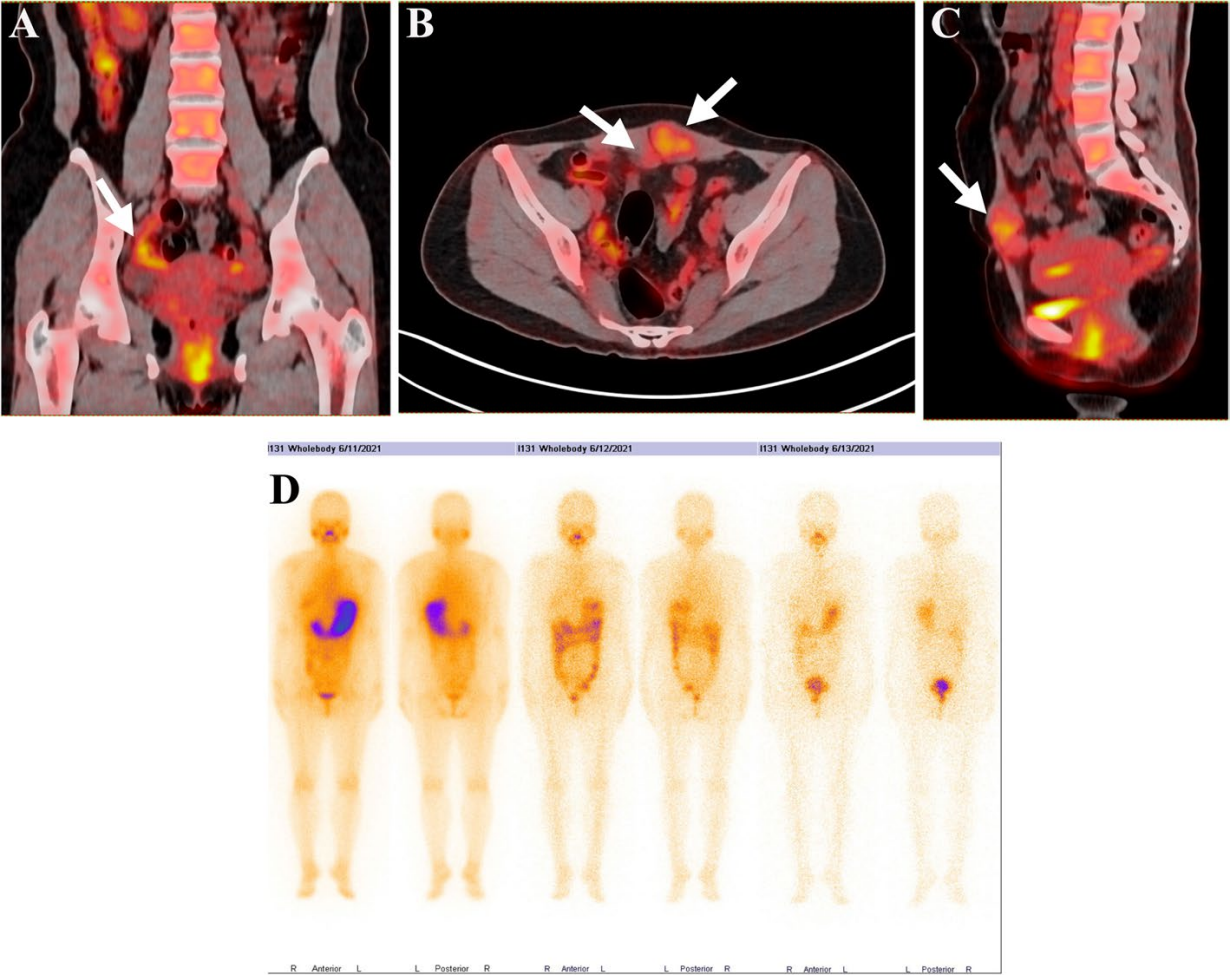
**A**, **B**, **C** PET-CT revealed the increased FDG uptake in abdominopelvic cavities. **D** No residual I.^131^uptake

A 38-year-old, para 1 gravida 5 woman has a long history of recurrent cysts on the right ovary. In 2005, she underwent the first ovarian cystectomy and the postoperative pathology demonstrated OMCT. In 2011, the second cystectomy was performed during a cesarean section and the pathological diagnosis was SO. In 2014, the patient received surgery again for a slowly growing cyst. In addition to the ovarian cyst, scattered cysts on the peritoneum and omentum were observed and removed intraoperatively, which were found to consist entirely of benign thyroid tissue pathologically. In August 2020, she was admitted to our hospital for the third relapse. PET-CT revealed a 2 cm mass on the right adnexal, multiple soft tissue masses in the left rectus abdominis and the peritoneum (Fig. 1A, B, C). Thyroid function tests and the ultrasound was normal. After multidisciplinary consultation and informed patient consent, laparoscopic total hysterectomy, bilateral salpingo-oophorectomy, omentectomy, pelvic lymphadenectomy, paraaortic lymphadenectomy and excision of visible lesions was performed to achieve complete resection. The histopathology confirmed recurrent HDFCO with pelvic-abdominal dissemination. Two of the 3 paraaortic lymph nodes were positive, and all 15 pelvic lymph nodes were negative. The patient was subsequently treated with thyroidectomy and 2-course I^131^ ablation in January 2021 and June 2021, respectively. An I^131^ body-wide uptake scan after therapy showed no residual radioactive uptake (Fig. 1D). On regular outpatient follow-up, the patient is currently disease free (Supplemental Table 1).

## Material and methods

See the Data Supplement.

## Results

### Histopathological evaluation: the developing spectrum of HDFCO

HDFCO is a newly recognized neoplasm that first described in 2008, characterized by extraovarian spreading of thyroid elements with a nonneoplastic histologic appearance [1]. The diagnosis of HDFCO is difficult and usually cannot be made until the neoplasm spreads beyond the ovary. Existing evidence emphasizes histological benignity and extraovarian spreading in the diagnosis of HDFCO.

**Fig. 2:**
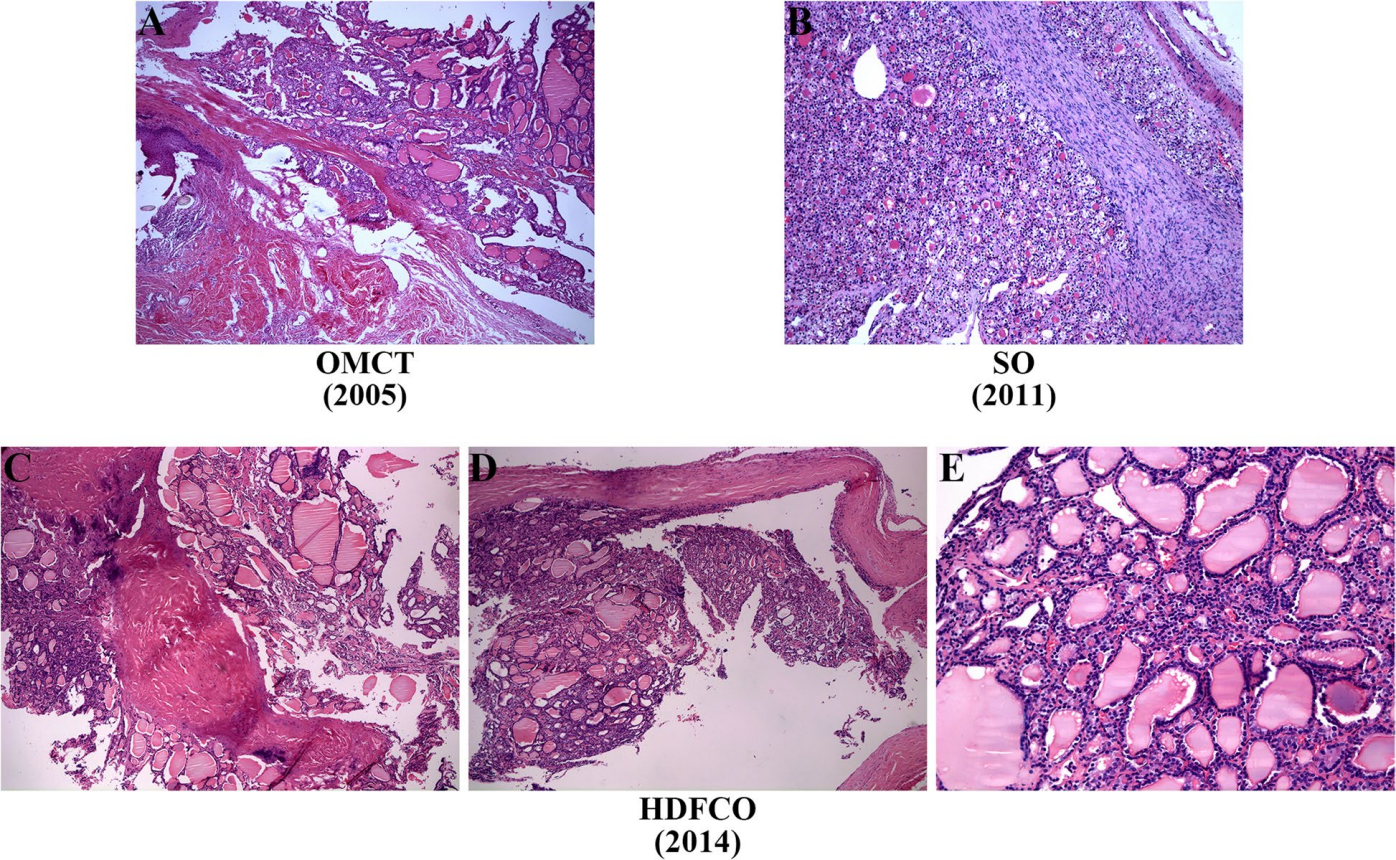
**A** Squamous epithelium, hair, adipose tissue, fibrous connective tissue, and thyroid tissue (× 50). **B** Nodular-distributed single benign thyroid component with focal hyperplasia of follicular epithelium (× 100). **C** Nodular-distributed single thyroid element and giant follicular in the right ovary (× 50). **D** The surface of peritoneum was involved (× 50). **E** No atypia, papillary architecture, ground glass nuclei, nuclear grooves, and mitosis (× 200)

**Fig. 3:**
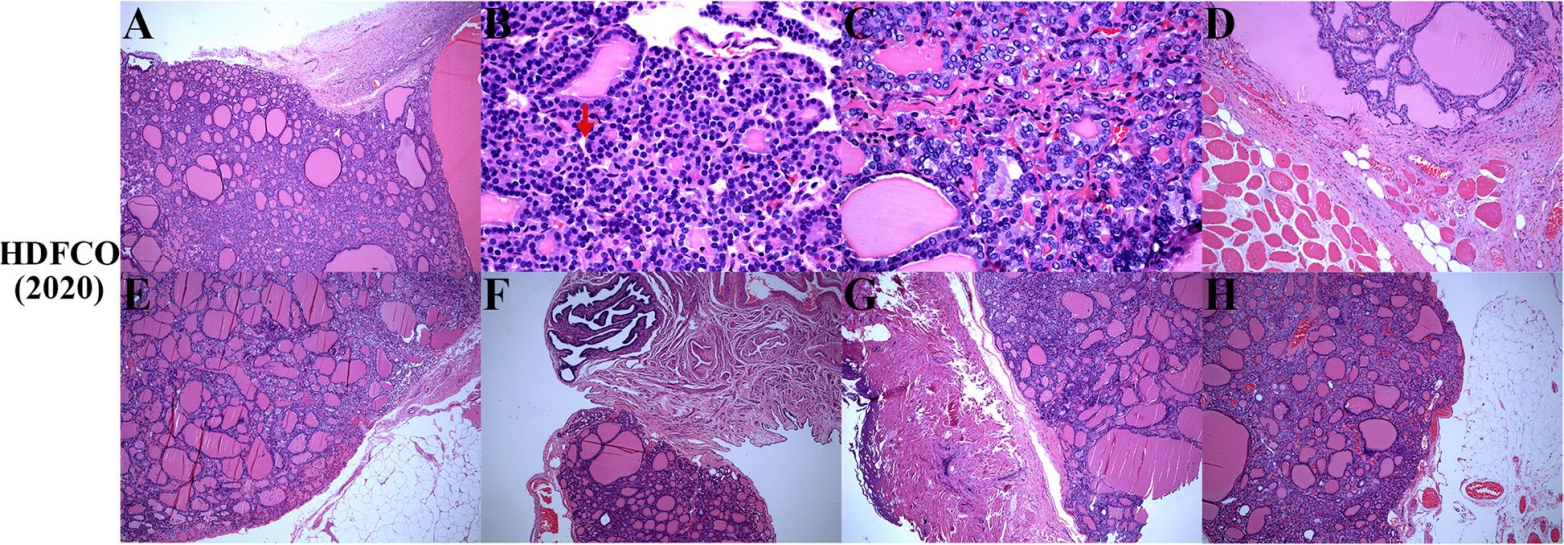
**A** Thyroid components replaced the ovarian cortex and medulla, locally nearly breached the surface (× 50). **B** Minimal mitotic activity (arrow, × 400). **C** Scattered ground glass nuclei (× 400). **D** The rectus abdominis was partially destroyed by tumor tissue (× 100). **E** The lymph node was totally replaced (× 50). **F**, **G**, **H** The tumor spread to the surface of right fallopian tube, rectum, and omentum with well-defined margins (× 50)

**Fig. 4:**
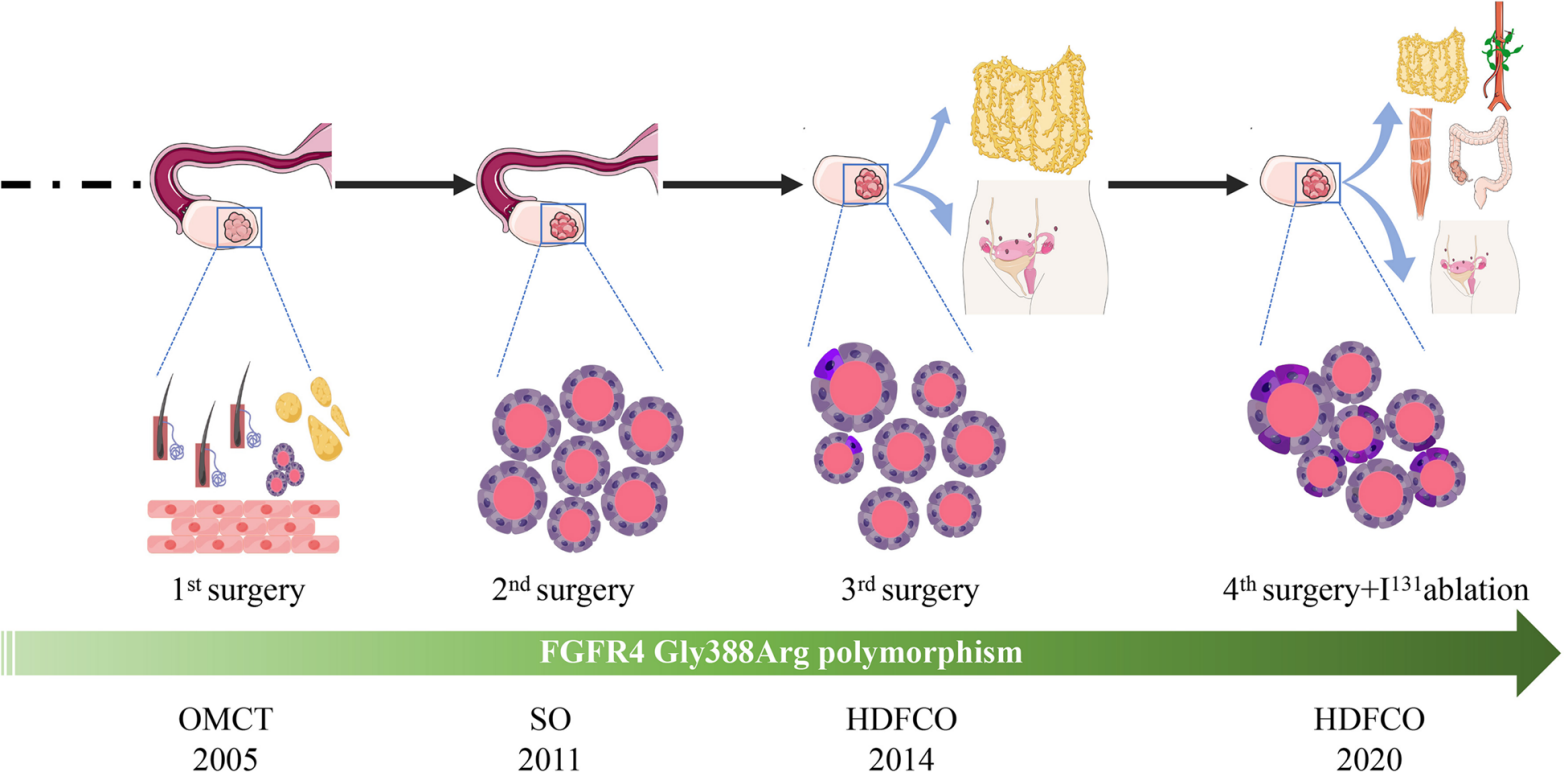
The developing spectrum of HDFCO

On histological evaluation, existence of tissues from different germ layers such as squamous epithelium, adipose tissue and small account of thyroid component confirmed OMCT in 2005 (Fig. 2A). Tumor in 2011 confined to the ovary was initially identified to be SO considering the sole presence of benign thyroid tissue (Fig. 2B). The subsequent recurrence and pelvic-abdominal involvement in 2014 (Fig. 2C) and 2020 (Fig. 3) indicated the tumor has developed to HDFCO. As showed, the involvement of adjacent organs by HDFCO was not invasive but limited to the surface (Figs. 2D and 3F, G, H). While the tumor behaved more aggressively after 3 times relapses, which was manifested by the appearance of mitosis (Fig. 3B) and ground glass nuclei (Fig. 3C), the tendency to breach the ovary, the expansion of extraovarian spread, lymphatic involvement (Fig. 3E) and the destruction of muscle tissue (Fig. 3D). Histopathology revealed that this case was a HDFCO and showed a consecutive malignant transformation from OMCT to SO to HDFCO which we called a developing spectrum (Fig. 4).

### Molecular analysis: FGFR4 Gly388Arg polymorphism

**Fig. 5:**
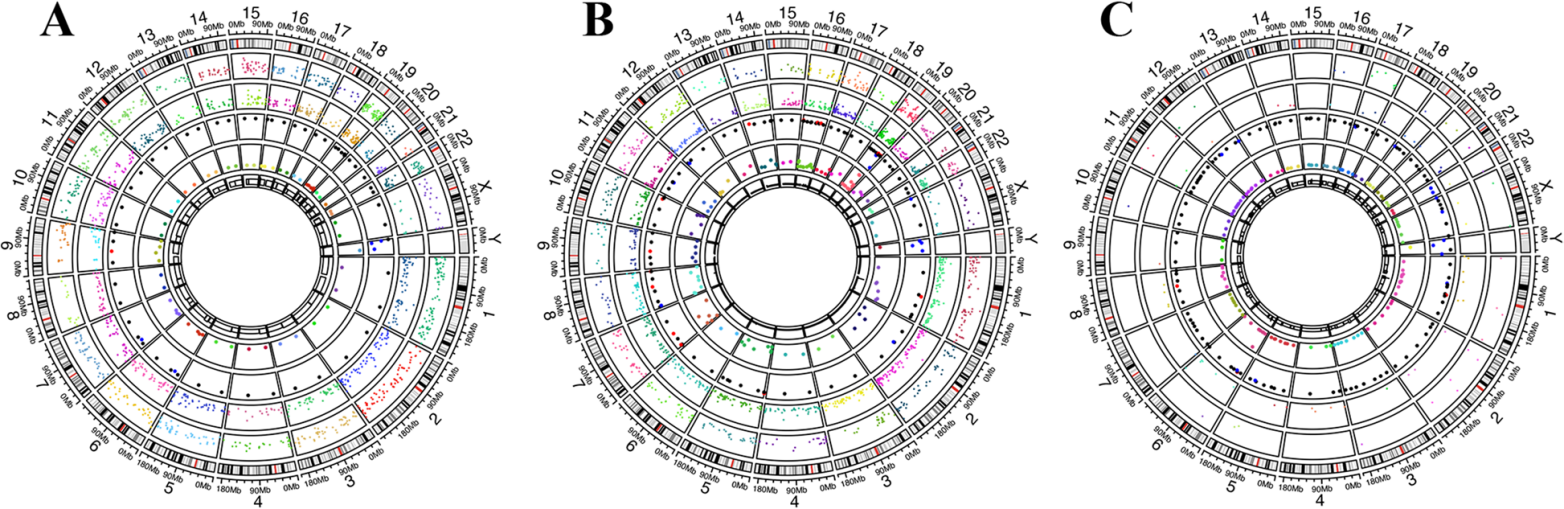
The results of whole-exome sequencing. **A** Primary tumor in 2011. **B** Primary tumor in 2020. **C** Extraovarian lesions in 2020

**Fig. 6:**
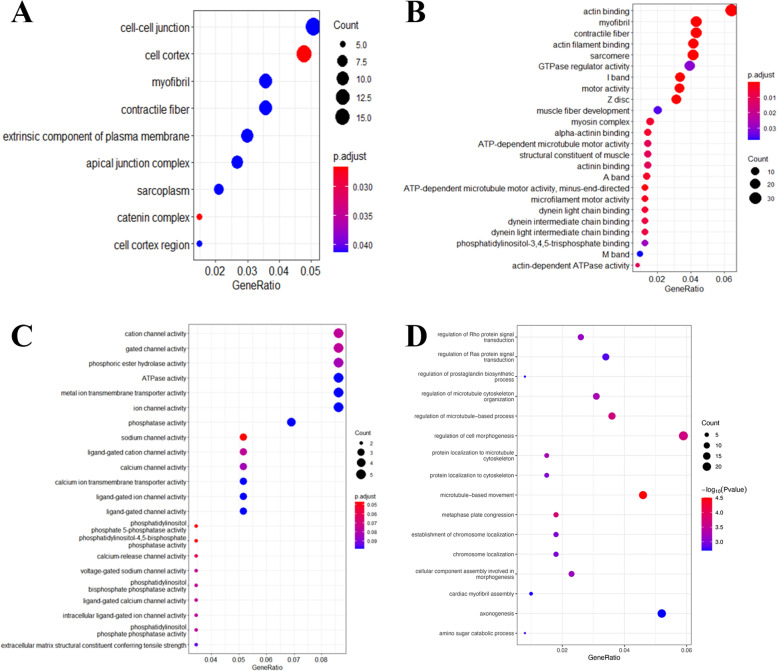
The GO and pathway enrichment analysis. **A**, **B**, **C** Somatic mutations. **D** Germline mutations

Little common somatic mutations were observed in three tumor samples, accounting for 0.1% of the total gene alterations. The primary lesions in 2011 and 2020 shared 5.7% common gene variations while the primary and extraovarian lesions in 2020 shared only 0.5% (Fig. 5). The gene ontology (GO) and pathway enrichment analysis revealed no clinically significant findings but extraordinary differences in the three samples (Fig. 6A, B, C). The total tumor mutation burden (TMB) of primary lesions in 2011, 2020 and extraovarian lesions in 2020 was 373, 585 and 62 Muts, respectively. Such diversity in somatic mutations reflected the molecular variety in the tumor progression and the heterogeneity between intra- and extra-ovarian lesions.

Interestingly, in terms of germline mutations, the patient harbored FGFR4 polymorphism (exon9: c.1162G > A: p.G388R), which was documented as a pathogenic alteration in ClinVar database. Additionally, the GO and pathway enrichment analysis showed an association between germline mutations and the RAS pathway (Fig. 6D), which implied that FGFR4 mutation may contribute to the malignant transformation. FGFR4 Gly388Arg polymorphism was also observed in her son, confirming the germline variant again.

### Literature review: clinical features of HDFCO

Totally 22 articles were identified from Pubmed. Except 18 cases originally described by Roth et al. in 2008, ten publications were considered eligible for our study comprising 11 cases of HDFCO [3–12]. (Supplemental Table 2) The median age at diagnosis is 32 years. The mean interval of recurrence is 17.6 years, and the median is 11 years, ranging from 6 to 48 years. None died of disease. Considering the relatively long recurrent interval and low mortality, HDFCO has a more favorable prognosis due to mild pathologic features and noninvasive extraovarian spread. The peritoneal involvement is the most common and earliest. While the systemic dissemination occurs after oophorectomy, including the liver, lungs, bone, cranial vault, and other sites.

Comparing to previously reported cases, the present case obviously behaved more aggressive, reflected by shorter recurrent intervals (3, 6 years respectively), penetration to ovarian serosa, destruction of the muscle tissue, extensive dissemination, and lymphatic invasion.

## Discussion

### The developing spectrum of HDFCO

Ovarian mature teratoma is a benign tumor originating from a single germ cell, containing a mixture of differentiated tissues derived from the three germ layers. Thyroid components can be observed in 5–20% cases of mature teratoma [4]. When thyroid tissue becomes the predominant element (> 50%) in teratomas, struma ovarii occurs. Struma ovarii is a monodermal variant of ovarian teratomas, accounting for 2–3% of mature ovarian teratomas [11]. Malignant transformation of struma ovarii is rare, occurring in 5% of all cases [5]. The histological and biological malignancy in ovarian struma is inequivalent. Struma-derived thyroid-type carcinomas are histologically malignant but the majority of them do not spread beyond the ovary. Cases having extraovarian dissemination with an innocuous appearance used to be diagnosed as malignant struma ovarii, metastatic struma ovarii, strumosis, or related terms [1].

In 2008, Roth et al. identified them as a new entity HDFCO and defined as ovarian struma disseminating beyond ovary but consisting of bland thyroid tissue [1]. As reported, HDFCO usually arises from SO, less frequently associated with OMCT [2]. However, a consecutive transformation from OMCT to SO to HDFCO was observed in our study, which was drawn as a developing spectrum of HDFCO.

Because of its rarity, HDFCO has elicited considerable interest and our understanding of its histopathologic characteristics has been highly improved recently. More studies are expected to promote our insights into molecular mechanism. Unlike traditional struma-derived thyroid-type carcinoma, HDFCO has benign histology and relatively indolent behavior, while the present case behaves more aggressive. An underlying mechanism is needed to be explored to elucidate such difference.

### FGFR4 Gly388Arg polymorphism may participate in the developing spectrum of HDFCO

Currently, the molecular characteristic of HDFCO is elusive for lack of common gene abnormalities seen in typical cervical thyroid cancer and struma-derived papillary carcinoma, such as BRAF, RAS, p53, and PPARg-PAX8 gene fusion [8, 10, 13, 14]. However, the whole-exome sequencing of our patient showed the germline FGFR4 Gly388Arg polymorphism in exon 9, which may provide new insights into the mechanism of HDFCO.

Fibroblast growth factor receptor 4 (FGFR4) is a member of receptor tyrosine kinases (RTKs), composed of an extracellular domain, a single transmembrane domain, and an intracellular tyrosine kinase domain [15]. FGFRs can bind to specific FGFs and participate in oncogenesis and tumor progression by regulating cell survival, proliferation, migration, and angiogenesis [16]. A recent study characterized the genetic landscape of FGFR alterations in 4853 tumors and recognized FGFR aberrations in 7.1% tumors [17]. Changes were most frequent in FGFR1 (3.5%), following FGFR3 (2.0%), FGFR2 (1.5%) and FGFR4 (0.5%). Multiple mechanisms can cause aberrant FGFR4 activation, including FGFR4 overexpression, FGF ligand overexpression, FGFR4 somatic hotspot mutations, and FGFR4 single nucleotide polymorphism (SNP) [15]. The SNP Gly388Arg at the transmembrane domain of FGFR4, a hot spot in RTKs, can enhance STAT3 activity by exposing a membrane proximal STAT3 binding site and finally accelerates cancer progression [18]. The FGFR4 Gly388Arg variant has been statistically proved to be related to poor survival, increased cancer susceptibility and nodal involvement, especially in breast and prostate cancer [19–21].

Furthermore, Henderson et al. recently proposed a “second hit” theory that the whole-genome and segmental homozygosity may be the primary genetic alterations in SO and extraovarian lesions were molecularly consistent with intraovarian struma without aggression until receiving a second hit [10]. The continuous clinicopathologic changes in this case support the “second hit” hypothesis. Additionally, the significant heterogeneity between intra- and extra-ovarian reflected by differences in somatic mutations and TMB also implies the existence of a second hit in the developing spectrum.

Briefly, it’s reasonable to conclude that the germline FGFR4 Gly388Arg polymorphism may contribute to the progression of HDFCO probably by functioning as a second hit in the developing spectrum. Therefore, FGFR4-specific inhibition is a considerable therapeutic strategy if the patient relapses again. As for her son, it is of great clinical significance to closely follow up potential malignancy, including lung cancer, prostate cancer, liver cancer and so on.

Meanwhile, it’s also worth noting that the patient was pregnant for 4 times after the first surgery, in 2007, 2011, 2012, 2015 respectively. The high HCG environment during pregnancy may also induce malignant transformation by stimulating TSH receptor in tumor tissues [22, 23].

## Supplementary Information

**Additional file 1.**

**Additional file 2:** **Supplemental Table 1.** Patient’s past surgical history. **Supplemental Table 2.** Literature review of HDFCO.

## Data Availability

The data used and/or analyzed during the current study are available from the corresponding author on reasonable request.

## Abbreviations

FGFR: Fibroblast growth factor receptor
HDFCO: Highly differentiated follicular carcinoma
OMCT: Ovarian mature cystic teratoma
SO: Struma ovarii
PET-CT: Positron emission tomography-computed tomography
TMB: Tumor mutation burden
SNP: Single nucleotide polymorphism
HCG: Human chorionic gonadotropin
TSH: Thyroid-stimulating hormone
