# Supplementary material for: A rare case of highly differentiated follicular carcinoma in ovary with FGFR4 Gly388Arg polymorphism: a case report and literature review
Source: J Ovarian Res. 2022 Jun 14;15:71. doi: 10.1186/s13048-022-01007-y (PMC9195278; doi:10.1186/s13048-022-01007-y)
Supplement: Supplementary file 1 — Additional file 1. [file 13048_2022_1007_MOESM1_ESM.docx]

**A rare case of highly differentiated follicular carcinoma in ovary with FGFR4 Gly388Arg polymorphism: a case report and literature review**

Bao, et al

**Material and Methods**

**Sample collection and** **histological validation**

Tumor samples from all four operations were obtained for investigation. Formalin fixed, paraffin embedded (FFPE) sections of tumor tissues were stained with hematoxylin and eosin(H&E) and reviewed ﻿in consultation by the authors and other experienced pathologists in our institution. ﻿

The tumors were classified as biologically malignant if the ovarian tumor spread beyond the ovary or had penetrated to the ovarian serosa and was on the surface, or recurred, regardless of the earlier findings in the ovary (1, 2). ﻿The criteria for histologic malignancy and classification were in accordance with those of the World Health Organization and other major reference works (2-5).

**Whole-exome sequencing**

FFPE sections of all operation samples were firstly subjected to H&E staining to detect the tumor cell content. Only samples with a tumor percentage of ≥20% were qualified for subsequent analysis. Finally, whole-exome sequencing was performed on the tumor specimens from 2011 and 2020 to comprehensively evaluate gene alterations. The bioinformation analysis was as carried out following the procedure described in previous work (6).

**Droplet digital PCR**

Following the whole-exome sequencing, droplet digital PCR was performed on the peripheral blood samples from the patient’s son to detect targeted germline gene mutation.

**Literature review**

We searched the PubMed database from 2008 to September 2021 for all cases of HDFCO, using different combinations of keywords ‘struma ovarii’, ‘highly differentiated follicular carcinoma’, ‘malignant’ and ‘ovarian’ without any restriction. ﻿Appropriate publications were firstly evaluated by checking the titles and abstracts. Full text review was then performed to confirm the pathological diagnosis of HDFCO and to exclude duplicate cases. The references of all finally selected publications were also checked for additional studies that might have been missed in the initial search. The original data on clinical features and survival were collected from selected studies.

**References**

1. Devaney K, Snyder R, Norris HJ, Tavassoli FA. Proliferative and histologically malignant struma ovarii: a clinicopathologic study of 54 cases. Int J Gynecol Pathol. 1993;12(4):333-43.

2. Robboy SJ, Shaco-Levy R, Peng RY, Snyder MJ, Donahue J, Bentley RC, et al. Malignant struma ovarii: an analysis of 88 cases, including 27 with extraovarian spread. Int J Gynecol Pathol. 2009;28(5):405-22.

3. Garg K, Soslow RA, Rivera M, Tuttle MR, Ghossein RA. Histologically bland "extremely well differentiated" thyroid carcinomas arising in struma ovarii can recur and metastasize. Int J Gynecol Pathol. 2009;28(3):222-30.

4. Roth LM, Miller AW, 3rd, Talerman A. Typical thyroid-type carcinoma arising in struma ovarii: a report of 4 cases and review of the literature. Int J Gynecol Pathol. 2008;27(4):496-506.

5. Hedinger C WE, Sobin LH. Histological Typing of Thyroid Tumours. 2nd ed. Berlin: Springer Verlag; 1998.

6. Yang N, Li Y, Liu Z, Qin H, Du D, Cao X, et al. The characteristics of ctDNA reveal the high complexity in matching the corresponding tumor tissues. BMC Cancer. 2018;18(1):319.
