# Supplementary material for: A rare case of highly differentiated follicular carcinoma in ovary with FGFR4 Gly388Arg polymorphism: a case report and literature review
Source: J Ovarian Res. 2022 Jun 14;15:71. doi: 10.1186/s13048-022-01007-y (PMC9195278; doi:10.1186/s13048-022-01007-y)
Supplement: Supplementary file 2 — Additional file 2: Supplemental Table 1. Patient’s past surgical history. Supplemental Table 2. Literature review of HDFCO. [file 13048_2022_1007_MOESM2_ESM.docx]

**A rare case of highly differentiated follicular carcinoma in ovary with FGFR4 Gly388Arg polymorphism: a case report and literature review**

Bao, et al

Supplemental Tables 1 and 2

***Supplemental Table 1: Patient’s past surgical history***

| Year | Diagnosis | Size of nodules(cm) | Surgery | Sites of dissemination | Thyroidectomy | RAI-131 | | ﻿Follow-up |
| --- | --- | --- | --- | --- | --- | --- | --- | --- |
| 2005 | OMCT | NA | right ovarian cystectomy | - | - | | - | NED (8 months) |
| 2011 | ﻿SO | NA | right ovarian cystectomy | ﻿- | - | | - |  |
| 2014 | HDFCO | 2 | ﻿right ovarian cystectomy+lesions resection | peritoneum, omentum | No | | No |  |
| 2020 | HDFCO | 5 | TAH+BSO+PLN+PALN+omentectomy+lesions resection | peritoneum, para-aortic lymph nodes, omentum, the left rectus abdominis, the rectum | Yes | | Yes |  |

Abbreviations: NA=not available; PLN=pelvic lymphadenectomy; PALN=paraaortic lymphadenectomy; NED = no evidence of disease

***Supplemental Table 2: Literature review of HDFCO***

| Authors | Year | Age at detection | Interval of recurrence (year) | Primary surgery | Sites of dissemination | Treatment after recurrence | | | ﻿Follow-up |
| --- | --- | --- | --- | --- | --- | --- | --- | --- | --- |
|  |  |  |  |  |  | ﻿Lesions resection | ﻿Thyroidectomy | ﻿ RAI-131 |  |
| Carey et al | 2014 | 39 | 31 | TAH+BSO | peritoneum, epicardium | Yes | Yes | Yes | LWD |
| Wei S, et al | 2015 | 35* | NA | lesions resection | ﻿fallopian tube,﻿ urinary bladder, pelvic wall | ﻿ NA | NA | NA | NED (17 years) |
| ﻿Ranade et al | 2015 | 37 | 6,18 | ﻿ extent unknown | peritoneum,colon,liver,spleen,lung | Yes | Yes | Yes | LWD |
| ﻿Liu et al | 2017 | 42 | 30 | TAH+BSO | Peritoneum, colon, ﻿adrenal gland | Yes | No | No | LWD |
| ﻿Riggs et al | 2018 | 32 | 11 | RSO | Peritoneum, ﻿uterus | Yes | No | No | NED (6 months) |
| ﻿Tsukada et al | 2019 | 23 | 16 | LSO | Peritoneum | Yes | Yes | Yes | LWD (2 years) |
| Dobi et al* | 2019 | 52 | NA | TAH+BSO+﻿ rectosigmoid resection and anastomosis+﻿left pelvic and common iliac lymph node dissection | ﻿ Peritoneum, uterus, sigmoid colon, pelvic wall | No | No | No | NED (1 year) |
| ﻿Henderson et al. C1 | 2020 | 23 | 48 | extent unknown | ﻿﻿the right face, multiple osseous structures, liver, heart | Yes | Yes | Yes | NED (18 months) |
| ﻿Henderson et al. C2 | 2020 | 22 | 9 | RSO | Peritoneum | NA | NA | NA | NA |
| ﻿Li et al** | 2021 | 29 | 10 | ﻿bilateral ovarian cystectomy | Peritoneum | Yes | No | No | NED |
| ﻿Roth et al | 2021 | 20s | 6,9 | ﻿right ovarian cystectomy | NA | No | Yes | Yes | LWD (12 years) |

Abbreviations: TAH=total hysterectomy; BSO=bilateral salpingo oophorectomy;﻿ LSO=left salpingo oophorectomy; ﻿RSO=right salpingo oophorectomy; LWD = living with disease; ﻿NED = no evidence of disease; NA=not available; ﻿RAI-131 = radioactive iodine-131

* ﻿The tumor had already spread at the time of its original detection. ** The tumor was found during pregnancy
